# Survivin Expression and Prognostic Significance in Pediatric Malignant Peripheral Nerve Sheath Tumors (MPNST)

**DOI:** 10.1371/journal.pone.0080456

**Published:** 2013-11-26

**Authors:** Rita Alaggio, Riccardo Turrini, Daniela Boldrin, Anna Merlo, Claudio Gambini, Andrea Ferrari, Patrizia Dall'Igna, Cheryl M. Coffin, Annalisa Martines, Laura Bonaldi, Gian Luca De Salvo, Paola Zanovello, Antonio Rosato

**Affiliations:** 1 Department of Medicine, University of Padova, Padova, Italy; 2 Veneto Institute of Oncology IOV-IRCCS, Padova, Italy; 3 Servizio di Anatomia ed Istologia Patologica, Istituto Giannina Gaslini IRCCS, Genova, Italy; 4 Oncologia Pediatrica, Fondazione IRCCS, Istituto Nazionale dei Tumori (INT), Milano, Italy; 5 Department of Pediatrics, Section of Pediatric Surgery, University of Padova, Padova, Italy; 6 Department of Pathology, Microbiology, and Immunology, Vanderbilt University, Nashville, Tennessee, United States of America; 7 Department of Surgery, Oncology and Gastroenterology, University of Padova, Padova, Italy; Ohio State University Comprehensive Cancer Center, United States of America

## Abstract

Malignant peripheral nerve sheath tumors (MPNST) are very aggressive malignancies comprising approximately 5–10% of all soft tissue sarcomas. In this study, we focused on pediatric MPNST arising in the first 2 decades of life, as they represent one the most frequent non-rhabdomyosarcomatous soft tissue sarcomas in children. In MPNST, several genetic alterations affect the chromosomal region 17q encompassing the *BIRC5/SURVIVIN* gene. As cancer-specific expression of survivin has been found to be an effective marker for cancer detection and outcome prediction, we analyzed survivin expression in 35 tumor samples derived from young patients affected by sporadic and neurofibromatosis type 1-associated MPNST. Survivin mRNA and protein expression were assessed by Real-Time PCR and immunohistochemical staining, respectively, while gene amplification was analyzed by FISH. Data were correlated with the clinicopathological characteristics of patients. Survivin mRNA was overexpressed in pediatric MPNST and associated to a copy number gain of *BIRC5*; furthermore, increased levels of transcripts correlated with a higher FNCLCC tumor grade (grade 1 and 2 vs. 3, p = 0.0067), and with a lower survival probability (Log-rank test, p = 0.0038). Overall, these data support the concept that survivin can be regarded as a useful prognostic marker for pediatric MPNST and a promising target for therapeutic interventions.

## Introduction

Malignant peripheral nerve sheath tumors (MPNST) are highly aggressive cancers that comprise approximately 5–10% of all soft tissue sarcomas. Less than 20% of cases are diagnosed in the first two decades of life, although they represent one the most frequent non-rhabdomyosarcomatous soft tissue sarcomas in children [1], [2]. A substantial fraction (21–67%) of MPNST arises in patients affected by neurofibromatosis type 1 (NF1) due to malignant transformation of neurofibromas [3]. Despite progress in the treatment of pediatric sarcomas, MPNST are poorly responsive to chemotherapy and radiotherapy, and their 5-year survival rate ranges from 82% for patients in Intergroup Rhabdomyosarcoma Study (IRS) stage I, to 26% for those affected by metastatic disease (IRS stage IV) [4]. For MPNST occurring in patients with NF1, the rate of response to chemotherapy is lower (17.6% *vs*. 55.3% in patients without NF1), and the outcome is worse with a 5-year overall survival of 32% [4]. Unfortunately, histological features and tumor grade do not predict the clinical behavior and metastatic potential for MPNST [5]. It is unclear whether the poor correlation between morphology and outcome is related to intrinsic characteristics of the neoplasm, such as the tendency to spread along nerves, and whether activation of molecular pathways that are not reflected in specific phenotypic features, such as apoptosis, mitoses, or pleomorphism, are relevant to prognosis.

The molecular mechanisms responsible for malignant transformation of neurofibromas in NF1, and those involved in tumor progression for the development of sporadic or NF1-associated MPNST, are largely unknown. Neurofibromatosis type 1 is caused by a mutation in the *NF1* suppressor gene, which is located in chromosome band 17q11 and codes for neurofibromin, a protein inhibiting p21-RAS [3]. Biallelic *NF1* inactivation is required for progression toward MPNST, but additional genetic alterations are also necessary, most likely involving genes that regulate the cell cycle [6]. Recent studies indicate that *TP53* is mutated in approximately 75% of MPNST [7], while deletions of the *CDKN2A* gene, which codes for p16^INK4A^ and p19^ARF^, are found in about 50% of neoplasms [8]. Among the numerous genetic aberrations reported in MPNST, which involve multiple losses on chromosome regions 1p, 9p, 11, 12p, 14q, 18, 22q, X and Y, and gains in chromosomal regions 7p and q, 8q and 15q, the recurrent gain of the distal part of chromosome 17q has been associated with a poor outcome [9]. Interestingly, the *BIRC5/SURVIVIN* gene (baculoviral inhibitor of apoptosis repeat-containing 5/survivin), a member of the inhibitor of apoptosis (IAP) family, is located in the same 17q25 region and it is a strong candidate target gene for amplification in adult MPNST [10]–[12].

Survivin blocks apoptosis induced by a variety of pro-apoptotic stimuli, including chemotherapy and radiation, in many malignancies [13]. Furthermore, increased levels of survivin are associated with a poor prognosis in numerous tumors [14]–[18], although some reports indicate that an elevated expression of survivin splice variants may represent a favorable prognostic marker in some cancers [19]. High survivin mRNA and protein levels seem to be significantly correlated with a poor prognosis in sarcomas, although there are few studies focusing on specific sarcomatous subtypes, sometimes with divergent results [20], [21]. Recent studies have suggested that *BIRC5/SURVIVIN* may represent a potential candidate gene associated with an unfavorable prognosis in MPNST in adult patients [10], [11].

Here, we report the analysis of survivin gene and protein expression, and gene copy number in a population of pediatric patients affected by sporadic and syndromic MPNST, and show that survivin perform well as prognostic marker for such tumors.

## Materials and Methods

### Ethics Statement

Samples and clinical data were collected for patients enrolled in the RMS'88 and RMS'96 AIEOP (Italian Association of Pediatric Hematology and Oncology) cooperative protocols. At the time of protocol submission, Ethical Committees were not present in Italy (they were established later according to the guidelines contained in the Ministerial Decree 18/03/1998); therefore, protocols were reviewed and approved by the “Commissione per la Sperimentazione Clinica dei Farmaci” of Complesso Clinico-Ospedaliero of Padua University, which deputized for such ethical aspects. Moreover, according to the laws in force at the time of enrollment, a written informed consent was not required and an oral informed consent from parents or caretakers was documented in the clinical records of the minors.

### Case selection

The surgical pathology files and consultation files at the Pathology Unit of Padua University and Primary Children's Medical Center (Salt Lake City, Utah) were screened for cases diagnosed as MPNST from 1990 to 2007. Synovial sarcomas were excluded from this study by a combination of morphologic, immunohistochemical, and genetic analyses. Thirty-five tumors met criteria for MPNST [22]. Tumors were graded according to the FNCLCC system (French Federation of Cancer Centers Sarcoma Group [23]). Clinical features (sex, age, location, NF1 status), histological characteristics and follow-up data were fully available for 23 patients, and partially for the remaining 12.

### RNA extraction and real-time RT-PCR assay

Total RNA was extracted from 2 sections (10 µm) of formalin-fixed and paraffin-embedded (FFPE) specimen using PureLinkTM FFPE Total RNA Isolation Kit (Invitrogen, San Giuliano Milanese, Italy), following the manufacturer's specifications. Conversion of RNA into cDNA and quantification of gene expression were carried out as previously reported [24], [25] using commercial on-demand assays (Hs00153353_m1, *BIRC5*; Hs99999905_m1, *GAPDH*; Applied Biosystems, Foster City, CA). The expression of survivin in each sample was determined using the 2^−ΔΔCt^ method [26]. Nine normal samples of pediatric soft tissue specimens including nerves were used as controls.

### FISH analysis

FISH was carried out with BAC clone RP11-219G17 [10] (BAC PAC Resources, http://bacpac.chori.org), which includes *BIRC5*, the survivin-encoding gene, and a centromeric probe for chromosome 17 (CEP17) (Visys-Abbott, Downers Grove, IL, USA). The BAC probe was prepared from bacterial cultures using Qiagen-Plasmid Midi kit (Qiagen GmnH, Dϋsseldorf, Germany) and labelled by nick translation with SpectrumOrange-dUTP (Visys). Before being used for the analysis of tumor samples, the probe was assessed by PCR with primers specific for survivin gene (primer pair For 5′- GTG AAC GGA TAC CTC TCT ATA TGC TG-3′ and Rev 5′-CTG ACT ATC ACC GTT ACC AGA ACT G-3′ and the following conditions: denaturation 1 min at 94°C, annealing 2 min at 59°C and extension 3 min at 72°C for 35 cycles; the expected length of the amplified DNA was 949 bp), and by FISH on normal metaphases from PHA-stimulated peripheral blood mononuclear cells to check adequacy and consistency of hybridization signal. FISH was performed on 4 µm FFPE sections as previously described [25]. For each probe set, 100 non-overlapping nuclei were enumerated and results were reported as copy number of *BIRC5* and CEP17. The presence of three or more copies of *BIRC5* in more than 30% of cells analyzed was defined as *BIRC5* copy number gain, while amplification of *BIRC5* was defined as a ratio between number of signals of BAC probe *BIRC5*/CEP17>2 or, in case of polysomy of chromosome 17, as a *BIRC5* gene copy number mean higher than 6.

### Histology and immunohistochemistry (IHC)

Hematoxylin/Eosin (H&E)-stained sections were either available from the initial pathologic evaluation or additionally performed for the study. Immunohistochemical staining was performed on FFPE sections (4 µm) using an indirect immunoperoxidase-based technique (Bond polymer Refine detection, Vision Biosystem, Newcastle upon Tyne, UK) and a fully automated system (Bond-maX, Vision Biosystem). Sections were incubated with a rabbit anti-survivin polyclonal antiserum (diluted 1∶50, Novus Biological, Marlupo, Rome, Italy), followed by a secondary antibody labeled with horseradish peroxidase (HRP) and diaminobenzidine, and finally counterstained with haematoxylin. For each case, survivin immunostaining of neoplastic cells was examined in 2 slides of tumor tissue. For each slide, 5 fields at 40X were evaluated and the percentage of positive cells was calculated as the average of stained cells in the total of 10 fields examined. Samples were arbitrary scored positive when more than 10% of the cells reacted with the antibody. Controls were represented by the same samples used to detect survivin mRNA. Results were reviewed by two pathologists in a blinded manner. Samples had also been previously stained for smooth muscle actin (SMA), muscle-specific actin, epithelial membrane antigen, cytokeratin and S-100 protein.

### Statistical analysis

To evaluate the association between the level of survivin expression with clinicopathologic characteristics, the Fisher Exact test was applied. Survivin mRNA levels were divided in high and low values according to the median (75) fold change. Estimates of the distribution of overall survival were calculated using the Kaplan-Meier method, while survival was measured from diagnosis date until death or until the date of last contact if the patient was alive at last report. All deaths were counted as failures whether or not they were disease-related. The log-rank test was used to compare the survival curves of patient subgroups at univariate analysis. Two sided p-values less than 0.05 were considered statistically significant. All data analyses were performed using the SAS statistical package (SAS, release 9.2; SAS Institute Inc, Cary, NC).

## Results

### Clinical findings

Age at diagnosis ranged from 1 to 18 years (median 11) and male:female ratio was 1∶1. Fifteen out of 26 patients with known NF1 status were affected by neurofibromatosis type 1. Clinical information for the 23 patients with available follow-up are reported in Table 1. Follow-up ranged from 2.1 to 136.8 months (median 23.9 months). Among these patients, 5 underwent surgical excision of the mass as unique treatment, while surgery was followed by chemotherapy for 7, radiotherapy for 3 and both chemotherapy and radiotherapy for 5. One patient underwent initial biopsy followed by chemotherapy only (case 14). Treatment was unknown for 2 patients.

**Table 1:** Clinicopathological characteristics of patients.

Case	Site	Sex	Age	NF1	Stage	First line treatment	Follow-up
1	Maxillofacial	F	3	no	IRS III	CE + CT + RT	Alive, progressive disease
2	Paravertebral	F	2	yes	IRS III	CE	First complete remission
3	Axilla	M	14	yes	IRS II	CE	Third complete remission
4	Laterocervical	M	18	no	IRS III	CE	First complete remission
5	Head	M	9	-	-	-	Lost at follow-up
6	Abdominal	M	1	yes	IRS I	CE + CT	Died of other causes
7	Paraspinal	M	2	no	IRS III	-	Alive, waste after therapy
8	Arm	M	11	-	IRS I	CE	Alive
9	Laterocervical	M	12	yes	IRS III	CE + RT	DOD
10	Arm	F	4	yes	IRS II	CE + RT	First complete remission
11	-	M	-	yes	IRS III	CE + CT	Alive
12	Arm	M	14	no	IRS I	CE + CT	DOD
13	Laterocervical	M	14	no	IRS III	CE + CT + RT	DOD
14	Presacral	F	11	yes	IRS III	CT	DOD
15	Leg	M	7	no	IRS I	CE	First complete remission
16	Retroperitoneal	F	15	yes	IRS III	CE + CT + RT	DOD
17	Leg	F	3	no	IRS III	CE + CT	First complete remission
18	Supravescical (right-hand side)	F	14	no	IRS I	CE + CT + RT	DOD
19	Gluteus	F	0	no	IRS III	CE + CT	Died for toxicity
20	Vagina	F	3	yes	IRS IV	CE + CT + RT	DOD
21	Dorsolumbar paravertebral (right-hand side)	F	2	yes	IRS III	CE + CT	DOD
22	Lower extremity	M	18	yes	IRS II	CE + RT	Alive with recurrent disease
23	Right hemithorax	F	8	-	-	CE + CT	Alive in first-line therapy

CE, Conservative excision; CT, chemotherapy; RT, radiotherapy; DOD, died of disease.

### Pathological findings

Histopathological, immunohistochemical and molecular findings are summarized in Table 2. All tumors were nodular and some of them were unencapsulated. The maximum diameter ranged from 1.5 to 16 cm (mean 9.1 cm, data not shown). Histologically, 29 tumors were classified as classic MPNST, 4 as epithelioid, 1 as glandular and 1 as Triton tumor. The majority of tumors were densely cellular with scattered hypocellular myxoid foci; in 3 tumors hypocellular myxoid areas were prominent. In epithelioid MPNST, variable amounts of epithelioid cells in small aggregates were intermingled with more conventional areas (data not shown). Several foci of glandular differentiation with evidence of mucin along the luminal borders were found in one MPNST. The Triton tumor showed fascicles of spindle cells with scattered elongated rhabdomyoblasts with hyperchromic nuclei and eosinophilic cytoplasm. The mitotic rate ranged from 0 to 85 per 10 high power fields (HPF), with a median of 11/10 HPF. Mitoses were more frequent in the hypercellular areas. Foci of necrosis were present in 7 tumors. Eight tumors were FNCLCC grade 1, 10 grade 2 and 17 grade 3.

**Table 2:** Histological characteristics and survivin expression of patient samples.

Case no.	Histology	FNCLCC Grade	Mitotic score	Survivin IHC	*BIRC5* copy number	Survivin mRNA
1	classic	3	2	+n		9.0
2	classic	2	1	+n	Monosomic	22.7
3	classic	3	3	+ +nc		44.9
4	classic	1	1	+n		50.7
5	classic	3	3			146.2
6	classic	3	3	++n	Gain	138.6
7	classic	1	1	+n	Disomic	71.3
8	classic	1	1	+n	Disomic	65.6
9	classic	3	2	neg	Gain	162.6
10	glandular	3	3	+nc		296.7
11	classic	2	2	+n	Disomic	115.4
12	epithelioid	3	3	+n	Disomic	1983.7
13	Triton	3	2	++n	Gain	161.9
14	classic	3	2	+n	Disomic	5118.1
15	classic	2	1	+n		44.9
16	classic	2	2	neg		11693.8
17	classic	2	1	neg		46.1
18	classic	3	3	++nc	Gain	537.9
19	epithelioid	3	3			641.4
20	classic	3	2			277.0
21	classic	3	3	++n	Gain	1521.0
22	classic	2	2	+n	Gain	8.6
23	classic	3	2	++n		102.2
24	classic	1	1		Disomic	5.9
25	classic	1	1		Disomic	7.7
26	classic	2	1		Gain	75
27	classic	1	1		Disomic	1.3
28	classic	1	1			2.8
29	epithelioid	3	2		Disomic	17.8
30	classic	2	1			149.0
31	classic	2	1			67. 3
32	classic	3	2	++nc	Disomic	761.4
33	classic	1	1			69.6
34	classic	2	2	++n	Amplified	113.5
35	epitheliod	3	1	+nc	Disomic	0.1

Mitoses were scored according FNCLCC (score 1: 0–9/10 HPF; score 2: 10–19/10 HPF; score 3: ≥20/10 HPF). Survivin IHC. (+) low positivity; (++) high positivity; n. nuclear; nc. nuclear and cytoplasmatic. Survivin mRNA: values referred to fold changes (low level: <75 fold change; high level: ≥75 fold change).

**Figure 1:**
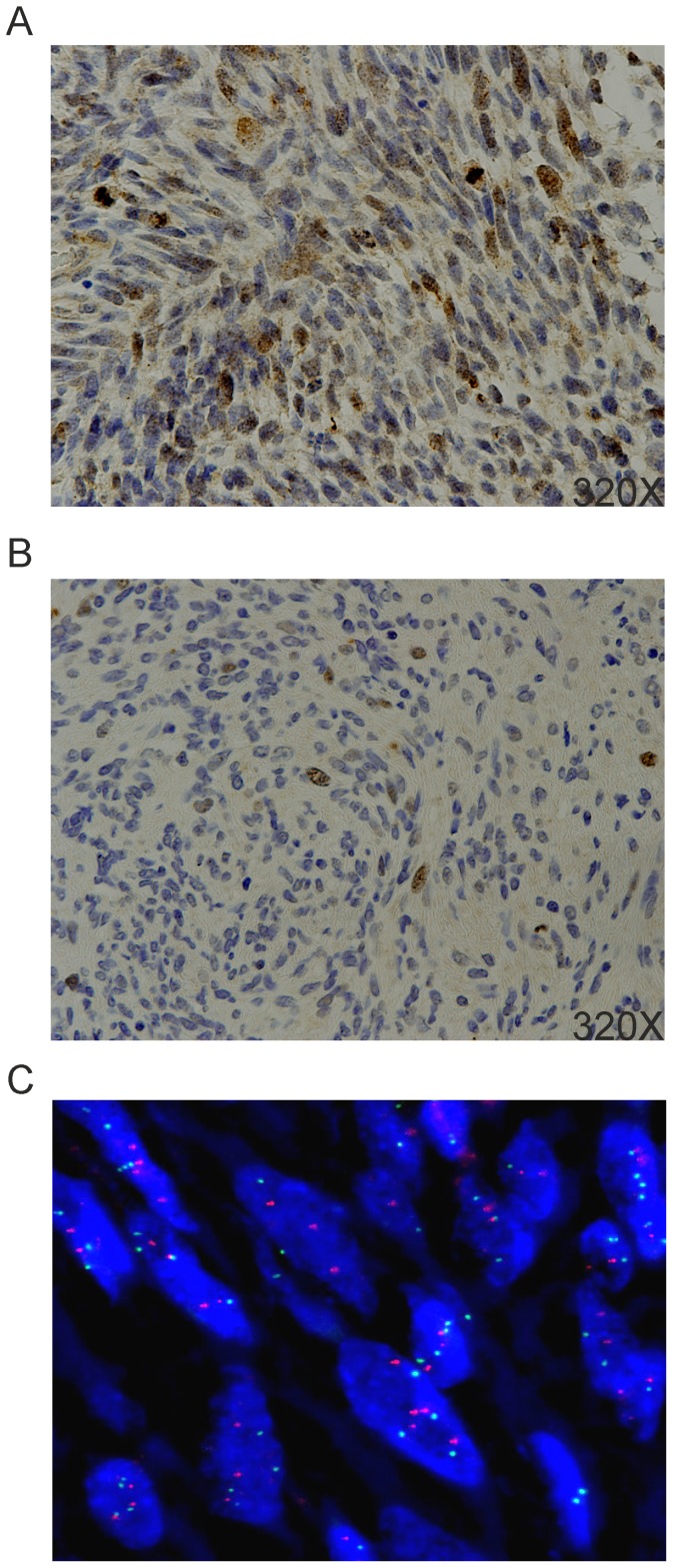
Survivin immunostaining and FISH analysis. IHC showing (A) a high positivity specimen (≥40%) with diffuse cells stained in the nucleus, and (B) a low positivity sample (<40%) with scattered neoplastic cells showing nuclear staining. (C) FISH analysis in a case of *BIRC5* copy number gain (red and green spots correspond to probes for *BIRC5* and CEP17, respectively; original magnification 100×).

IHC previously performed at the time of diagnosis showed S-100 reactivity in 61% of MPNST, with 13% of samples being immunoreactive in more than 20% of cells, and 48% of specimens displaying staining in scattered cells (data not shown); all tumors were nonreactive for cytokeratins and epithelial membrane antigen (data not shown). Myogenin was positive in rhabdomyoblasts in the Triton tumor (data not shown). Survivin immunostaining was evaluable in 23 out of 35 cases. Fifteen (65.2%) of such cases scored positive with an exclusive nuclear staining; 5 samples (21.8%) showed both nuclear and cytoplasmic positivity; 3 cases (13%) were negative and were not included in the subsequent correlation analysis because it could not be excluded that they were of sufficient good quality and conservation to rule out a false negativity. In fact, they also stained negatively with a series of different anti-survivin mAb or polyclonal antisera (data not shown), which conversely were capable of staining the other samples. Among positive samples, an intense stain in 40% of cells or more (high positivity) was present in 8 cases (40%), whereas the remaining 12 samples (60%) were positive in less than 40% of tumor population (low positivity), or presented a focal survivin staining (Figure 1A and B).

### Survivin expression analysis

Survivin mRNA was quantified by Real-Time PCR in all 35 MPNST cases. Survivin gene expression in tumors (fold-change median: 75; 95% CI for the median: 1–6104) was significantly higher than in control tissues (Mann-Whitney test, p = 0.0001); in particular, survivin mRNA was hardly detectable in controls (fold-change median: 1; 95% CI for the median: 0–36). Thus, high and low expression of the gene was defined as the level above or below the median, respectively (Table 2). Survivin mRNA levels correlated with survivin protein expression (Table 3, p = 0.0281); indeed, 15 samples analyzed by IHC had concordant survivin expression with both techniques, with strong positive staining (≥40%) associated with high mRNA levels (>75 fold-change), and weak staining with low mRNA values. Notably, higher levels of survivin mRNA were associated with high tumor grade (FNCLCC Grade 1 and 2 *vs*. Grade 3; p = 0.0067, Table 3) and in particular with a high mitotic score (score 1 *vs*. scores 2 and 3; p = 0.0001, Table 3).

**Table 3:** Correlation analysis.

Variable	Survivin mRNA		P value
	Low	High	
IHC			
Low (<40%)	8	4	0.0281
High (≥40%)	1	7	
FNCLCC Grade			
Grade 1–2	13	5	0.0067
Grade 3	4	13	
Mitotic score			
Score 1	13	2	0.0001
Score 2–3	4	16	
FISH			
Disomic/Monosomic	8	4	0.0281
Gain/Amplification	1	7	
Stage			
IRS I–II	4	4	0.6731
IRS III–IV	5	8	
Morphology			
Classic	15	14	0.6581
Other	2	4	

Survivin mRNA: values referred to fold changes (low level: <75 fold change; high level: ≥75 fold change).

FISH analysis for *BIRC5* gene was successfully carried out in 20 out of 26 cases tested. A gene copy number gain (Figure 1C) was present in 7 out of 20 samples (35%), while only one case (5%) resulted amplified. In the remaining 12 MPNST specimens, *BIRC5* was disomic in 11 cases (92%) and monosomic in one (8%) sample (Table 2). Interestingly, even though the number of samples analysed by both FISH and IHC was quite limited, most of the monosomic/disomic samples presented a low protein expression, and most of the sample with *BIRC5* gain/amplification displayed an increased staining by IHC. Moreover, *BIRC5* copy number significantly correlated with survivin mRNA levels (Table 3, p = 0.0281).

### Outcome data

**Figure 2:**
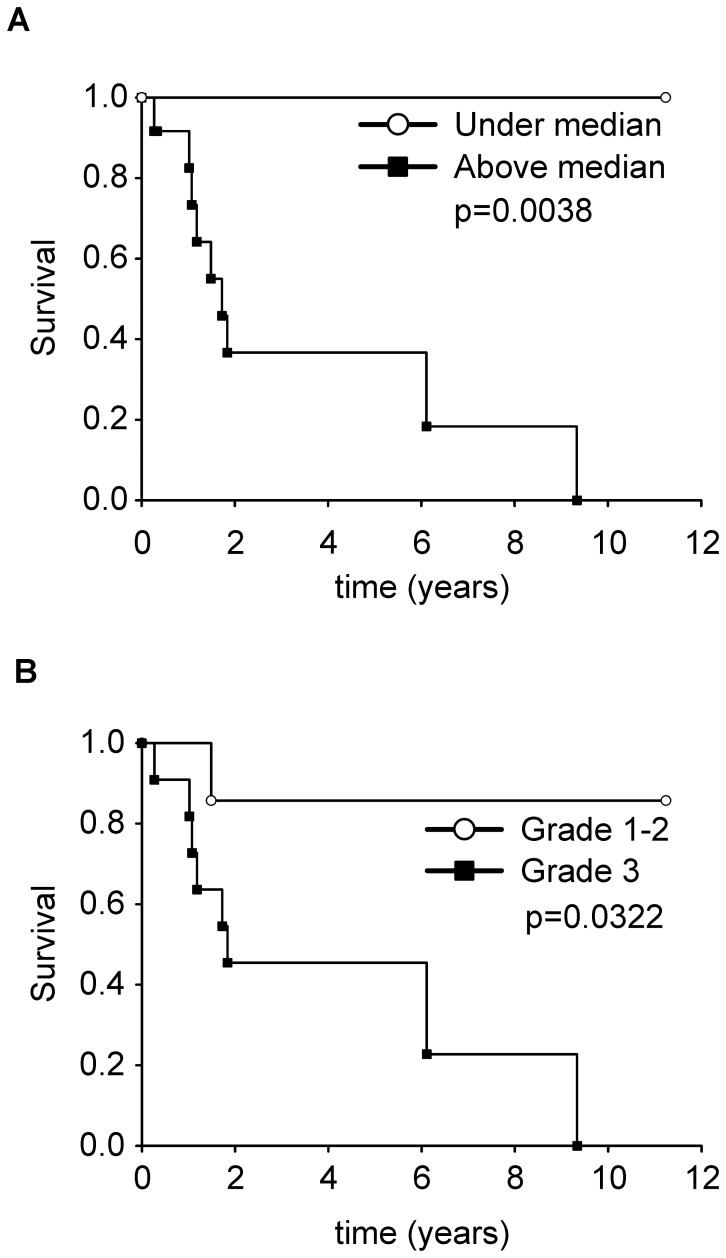
Survival analysis. (A) Kaplan-Meier survival curves of patients with survivin mRNA above (black squares) or under the median value (white circles). (B) Kaplan-Meier survival curves of patients with high FNCLCC grade (Grade 3, black squares) or low grade (Grade 1 and 2, white circles).

After a median follow up of 4 years (Inter Quartile Range: 1.2–11.3 years), 8 patients died of disease, 2 died of other causes, 12 were alive (3 with disease and 1 after relapse), and 1 was lost to follow-up. The survival analysis revealed an association between low levels of survivin mRNA and long-term survival (Figure 2A; Log-rank test, p = 0.0038). FNCLCC Grade 1 and 2 MPNST had a more prolonged survival when compared to FNCLCC grade 3 (Figure 2B; Log-rank test, p = 0.0322). There was no additional statistically significant relationship with other clinicopathological characteristics (stage, histological type, age, sex and NF-1 status; Table 3 and data not shown).

## Discussion

MPNST in children and adolescents may be sporadic, associated with NF1, or arising as a second malignancy after radiotherapy. Their clinical behavior is very aggressive, although in children younger than 7 years sporadic tumors have a better prognosis and appear to be more responsive to chemotherapy than those occurring in adults [4], [27]. The basis for these clinical differences and the biological features of MPNST in young patients are not well understood. Different gene profile studies have already described several genes and related protein products potentially accounting for prognostic or therapeutic roles in MPNST. In this regard, *CDK4* (Cyclin-dependant Kinase 4) gain/amplification and increased FoxM1 (Forkhead box protein M1) protein expression have been reported as predictors of poor survival in MPNST patients [28], while EGFR (Epithelial Growth Factor Receptor) overexpression is thought to play a role in MPNST progression and has been correlated with worse prognosis and clinical course [29]. Interestingly, Aurora kinase A is dramatically overexpressed in MPNST cell lines and its inhibition may limit tumor cell growth [30]; additionally, *in vitro* targeting of SOX9 (Sex-determining-region Y-box 9) by shRNA (short hairpin RNA) reduces MPNST cell survival and increases death [31]. Thus, it appears that several biochemical pathways are involved in the pathogenesis of MPNST. In such complex context, a number of independent studies revealed that the presence of alterations of chromosome 17, and in particular the overexpression of *BIRC5/SURVIVIN* gene, are frequently involved in these malignancies and might play a key role in their poor prognosis [9], [11], [32], [33]. Notwithstanding, all these studies analyzed heterogeneous samples from both pediatric and adult specimens, and most of them did not assess the impact of the target gene in survival.

On this ground, we decided to focus on *BIRC5/SURVIVIN* as a potential survival marker for MPNST prognosis in pediatric patients affected by MPNST. In our setting, while all tumors expressed survivin mRNA, there was no correlation between mRNA levels and NF1 status or tumor type, although high values were slightly more frequent in NF1-associated tumors (9/15) compared to sporadic cases (5/11). Thus, our data are in agreement with and enrich two previous studies carried out on a very limited number of MPNST (overall, 9 sporadic and 15 NF1-associated tumors) that included only two patients younger than 18 years [10], [32]. These works demonstrated a consistent up-regulation of survivin mRNA in MPNST compared to neurofibromas and schwannomas.

High levels of survivin mRNA were significantly associated with FNCLCC tumor grade 3 and a high mitotic rate. This might be explained by the central role of survivin in cell division, as its expression increases progressively in the cell cycle from the G1 to the G2 phase and during mitosis, when it regulates microtubular dynamics and separation of sister chromatids.

Survivin expression in low grade MPNST, both in the present and previous studies [10], [32], suggests that survivin up-regulation might represent an early event in malignant transformation, and that its increase might be involved in tumor progression in both syndromic and sporadic tumors. Since the prognostic role of a three-tier grading system in MPNST has been recently challenged [34], survivin expression levels might be advanced, among other criteria, as a parameter contributing to discriminate prognostic subsets of MPNST based on a two-tier histological grading system.

In addition to tumor grade, high levels of survivin mRNA were significantly associated with an aggressive clinical behavior, as 7/13 patients with high levels of transcripts died within 5 years from diagnosis whereas all patients with low survivin expression (9/9) were alive and in complete remission; such aspects are even better illustrated by Kaplan-Meier survival curves. These findings are in line with the adverse prognostic significance of elevated survivin mRNA and protein levels for soft tissue sarcomas in general, with a 15.5-fold increased risk of tumor-related death [35]. Accordingly, in a previous report on a smaller number of MPNST the unfavorable prognostic significance of survivin expression was hypothesized based on the relatively lower recurrence rate in survivin-negative MPNST [11].

An important feature for survivin molecular functions is its localization within the cell: in this regard, both nuclear and cytoplasmic staining have been correlated either to favorable or unfavorable outcome depending on the tumor histotype [36]. In our study, IHC staining localized survivin protein expression almost exclusively in the nucleus, with no difference between long- and short-term surviving patients. This observation implies that the prognostic significance of survivin localization is yet to be fully elucidated.

The biological mechanisms underlying survivin mRNA up-regulation are largely unknown. A recent study demonstrated a gain of distal 17q material in 16 out of 28 MPNST, and subsequent detailed FISH mapping analysis identified a 2 Mb commonly gained/amplified region at 17q25 where *BIRC5* gene is located [10]. For this reason, *BIRC5* has been considered one of the strong candidate target gene for amplification in these tumors. In our series, *BIRC5* gene was amplified only in 1 sample but a gene copy number gain was present in 35% of investigated tumors; moreover, FISH results correlated with mRNA levels and protein expression, thus indicating that in pediatric MPNST *BIRC5* gene copy number is likely involved in protein overexpression. Alternatively, overexpression of survivin might depend on regulation at the transcriptional level, as already reported in other malignancies [37]; in this case, *NF1* loss of function might promote survivin transcription by activation of nuclear factor-κb (NF-κB) via the phosphatidylinositol 3-kinase (PI3K)/Akt pathway or TCF-4/b-catenin pathway [38].

Overall, even though further studies are needed to precisely unveil the role and significance of survivin in MPNST, its large expression in pediatric cases underscores a potential role as target of therapeutic interventions, as reported preclinically for MPNST themselves or other malignancies [12], [39]. Additionally, the significant relationship between tumor grade and survivin levels may provide a potential biological basis for a two-tier grading system endowed with a prognostic significance in pediatric MPNST.
